# Incidence and risk of respiratory tract infection associated with specific drug therapy in pulmonary arterial hypertension: a systematic review

**DOI:** 10.1038/s41598-017-16349-7

**Published:** 2017-11-24

**Authors:** Zhichun Gu, Chi Zhang, Anhua Wei, Min Cui, Jun Pu, Houwen Lin, Xiaoyan Liu

**Affiliations:** 1 Department of Pharmacy, Renji Hospital, School of Medicine, Shanghai Jiaotong University, Shanghai, 200127 China; 2 Department of Pharmacy, Tongji Hospital, Tongji Medical College, Huazhong University of Science and Technology, Wuhan, 430030 China; 3 Department of Cardiology, Renji Hospital, School of Medicine, Shanghai Jiaotong University, Shanghai, 200127 China

## Abstract

Specific drug therapy has been proven to improve functional capacity and slow disease progression in pulmonary arterial hypertension (PAH), regretfully with the data on the risk of respiratory tract infection (RTI) associated with specific drug therapy being limited. Databases of Medline, Embase, Cochrane Library and the ClinicalTrials.gov Website were searched for randomized controlled trials (RCTs) that reported the RTI data of PAH-specific drug therapy in patients. The primacy outcome was assessed by employing a fixed-effects model. Totally, 24 trials involving 6307 patients were included in the analysis. PAH-specific drug therapy was not significantly associated with the increased risk of both RTI (19.4% vs. 21.1% RR 1.02, 95%CI 0.92–1.14, *P* = 0.69) and serious RTI (4.3% vs. 5.0% RR 0.99, 95%CI 0.77–1.26, *P* = 0.93) compared to placebo. The results were consistent across the key subgroups. No heterogeneity between the studies (I^2^ = 35.8% for RTI, and I^2^ = 0.0% for serious RTI) and no publication bias was identified. In conclusion, no significant increase in RTI had been found in PAH-specific drug therapy when compared with placebo. Whereas, RTI in PAH patients is still worthy of clinical attention.

## Introduction

Pulmonary arterial hypertension (PAH) is a fatal disease characterized by progressively increased pulmonary vascular resistance and pulmonary artery pressure, leading to right heart failure and death ultimately^1,2^. Although no cure exists for PAH nowadays, improved understanding of PAH pathobiological mechanisms resulted in the development of effective therapies^2^. Drugs for PAH-specific therapy, targeting the endothelial dysfunction and specific aberrant pathways, have been approved by the US Food and Drug Administration (FDA)^3^. So far, mainly 5 classes of specific drugs were applied for PAH, including prostanoids (PCAs), endothelin receptor antagonists (ERAs), phosphodiesterase type 5 inhibitors (PDE5 inhibitors), soluble guanylate cyclase stimulators (sGCs), and selective prostacyclin receptor agonists, each of which has been demonstrated to significantly improve exercise capacity, symptoms as well as hemodynamics, and to slow clinical worsening in clinical trials^4–8^. Nevertheless, infection is still an issue that cannot be neglected in PAH, which might cause progressive right cardiac failure and lead to clinical worsening. Although PAH-specific drugs are generally well tolerated, catheter-related blood stream infection (CR-BSI) was still confirmed to be a significant complication associated with the use of Intravenous prostanoid therapy^9,10^, and respiratory tract infection (RTI), was also reported as a significant factor leading to the deterioration of PAH^10^. In the SERAPHIN trial conducted on macitentan, the incidence of RTI and serious respiratory tract infection (SRTI) was 31.5% and 4.5% in the treatment group, respectively^5^. The class effects of PAH-specific drugs, including pulmonary vasodilatation and anti-proliferative effect of pulmonary artery, might be one of the factors inducing the increased risk of RTI^3^. Accordingly, for the drug safety, it is necessary to assess the incidence and risk of RTI in PAH patients using specific drugs.

## Results

### Study evaluation

**Figure 1:**
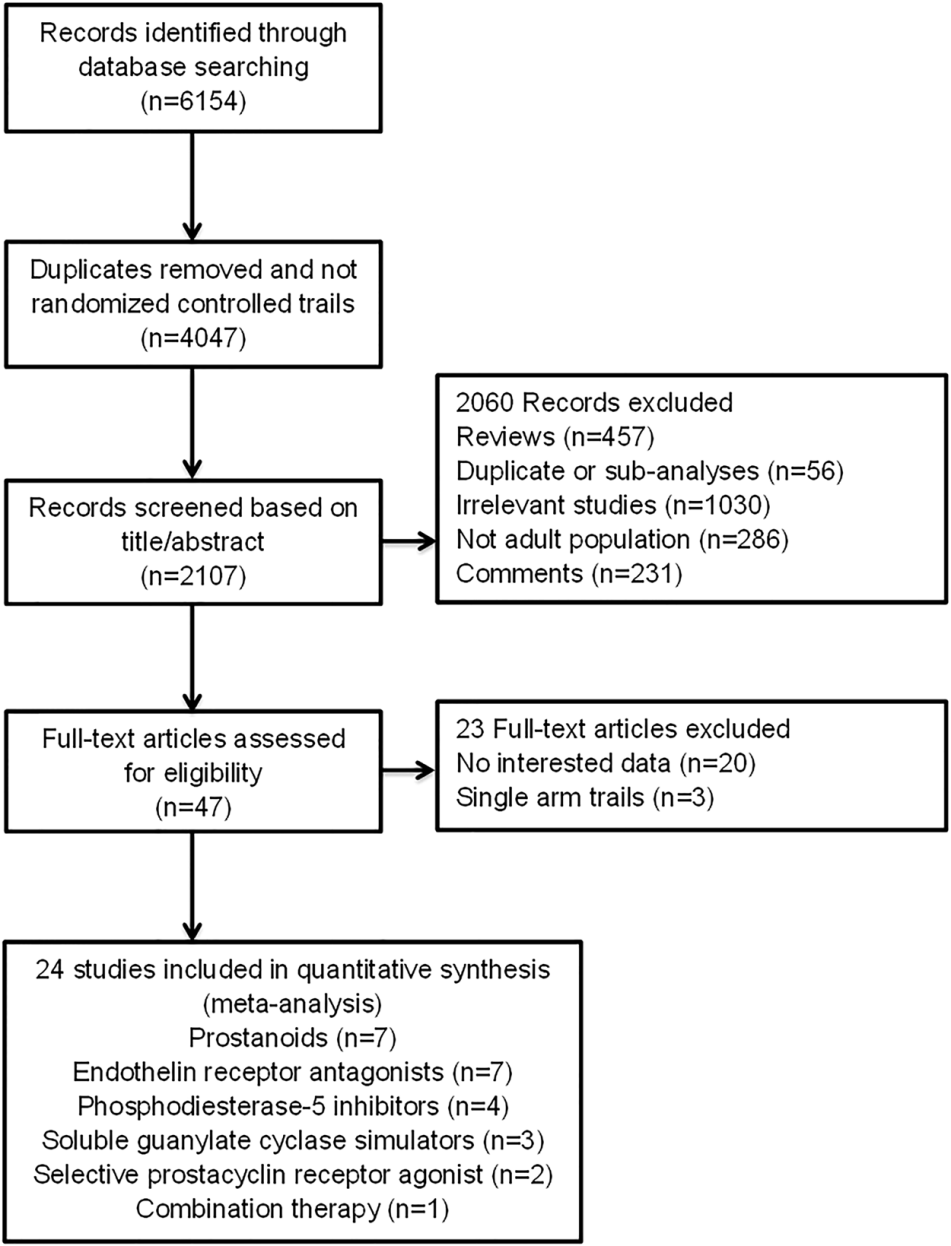
Flow diagram for the selection of eligible randomized controlled trials.

A total of 2107 records were identified from the initial database search. For various reasons through title and abstract screening, 2060 records were excluded. The remaining 47 records were full-text articles, of which 23 proved ineligible due to the unavailability of RTI data. Finally, 24 eligible RCTs were included in the analyses (Table S1, Fig. 1)^4–8,11–29^. The characteristics of included RCTs were summarized in Table 1. Publication year varied from 2005 to 2015, and trial duration ranged from 12 to 71 weeks. The size of the studies varied from 18 to 1152 patients, with the average of patients being 263 per study. Totally, 6307 PAH patients were enrolled, among which 4033 (63.9%) patients received PAH-specific drugs and 2274 (36.1%) patients received placebo. Of these 24 studies, 7 studies (1274 patients) concerned about PCAs, 7 (1453 patients) about ERAs, 4 (1058 patients) about PDE5 inhibitors, 3 (722 patients) about sGCs, 2 (1195 patients) about selective prostacyclin receptor agonist, and 1 (605 patients) about combination therapy of ERAs and PDE5. The included studies had low bias overall, with 4 trials at unclear risk of bias (Table S2). The quality of the evidence was considered to be high on this basis.

**Table 1:** Summarized Characteristics of Included Randomized Controlled Trials.

Source	Groups	Baseline therapy	N	Mean Age (y)	Female (%)	WHO FC (%)	Duration (weeks)	Etiology (%)	Outcome Measures
**PCA vs. Placebo**									
McLaughlin *et al*., 2006 (STEP)^14^	INH Iloprost	ERA	35	51.0	79.4	II (2)	12	IPAH (55),	RTI
						III (94)		APAH (45)	
	Placebo		32	49.0	78.8	IV (4)			
Hoeper *et al*., 2006 (COMBI)^15^	INH Iloprost	ERA	19	48.0	21.1	III (100)	12	IPAH (100)	RTI
	Placebo		21	56.0	23.8				
McLaughlin *et al*., 2010 (TRIUMPH)^16^	INH Treprostinil	ERA, or PDE5	115	55.0	80.9	III (98)	12	IPAH (56),	RTI, SRTI
						IV (2)		APAH (33)	
	Placebo		120	52.0	81.7			Others (11)	
Tapson *et al*., 2012 (FREEDOM-C)^17^	Oral Treprostinil	ERA, PDE5, or both	174	51.0	85.1	II (21)	16	IPAH (66),	RTI, SRTI
						III (76)		APAH (34)	
	Placebo		176	50.0	79.5	IV (3)			
Tapson *et al*., 2013 (FREEDOM-C2)^18^	Oral Treprostinil	ERA, PDE5i, or both	157	51.5	75.8	II (26)	16	IPAH (66),	RTI, SRTI
	Placebo		153	50.4	79.7	III (73)		APAH (34)	
Jing *et al*., 2013 (FREEDOM-M)^19^	Oral Treprostinil	Conventional therapy	151	37.8	72.0	II (33)	12	IPAH (75),	RTI, SRTI
	Placebo		77	42.5	75.0	III (66)		APAH (25)	
Hiremath *et al*., 2010 (TRUST)^4^	IV Treprostinil	Conventional therapy	30	30.0	63.3	III (100)	12	IPAH	SRTI
	Placebo		14	36.0	57.1				
**ERA vs. Placebo**									
Rubin *et al*., 2002 (BREATHE-1)^11^	Bosentan	Conventional therapy	144	48.7	79.2	III (92)	16	IPAH (70),	SRTI
	Placebo		69	47.2	78.3	IV (8)		APAH (30)	
Humbert *et al*., 2004 (BREATHE-2)^20^	Bosentan	PCA	22	45.0	77.3	III (76)	16	IPAH (82),	RTI, SRTI
	Placebo		11	47.0	54.5	IV (24)		APAH (18)	
Corte *et al*., 2014 (BPHIT)^21^	Bosentan	Conventional therapy	40	66.4	32.5	II (7)	16	FIIP-PH	SRTI
						III (43)		−100	
	Placebo		20	66.9	25	IV (50)			
McLaughlin *et al*., 2015 (COMPASS-2)^22^	Bosentan	PDE5	159	52.9	78.6	I (42)	16	IPAH (68), APAH (32)	RTI, SRTI
						II (58)			
	Placebo		174	54.7	73.1	IV (<1)			
ARTEMIS-PH^23^	Ambrisentan	Conventional therapy	25	68.0	20	NA	56	IPF-PH	RTI, SRTI
	Placebo		15	68.0	33.3			−100	
AMBER I^24^	Ambrisentan	Conventional therapy	17	63.0	47.1	NA	16	CTEPH	RTI, SRTI
	Placebo		16	59.0	62.5			−100	
Pulido *et al*., 2013 (SERAPHIN)^5^	Macitentan	PCA, PDE5, or no	492	45.1	77.4	II (52)	24	IPAH (56)	RTI, SRTI
						III (46)		APAH (44)	
	Placebo		249	46.7	73.9	IV (2)			
**PDE5 vs. Placebo**									
Galiè *et al*., 2005 (SUPER-1)^12^	Sildenafil	Conventional therapy	207	48.7	73.4	II (39)	12	IPAH (63)	SRTI
	Placebo		70	49.0	81	III (58)		APAH (27)	
Simonneau *et al*., 2008 (PACES)^25^	Sildenafil	PCA	134	47.8	82.1	II (25)	16	IPAH (79)	RTI, SRTI
						III (66)		APAH (21)	
	Placebo		131	47.5	77.4	IV (6)			
Galiè *et al*., 2009 (PHIRST)^6^	Tadalafil	ERA, or no	323	53.5	78	II (35)	16	IPAH (63)	RTI
	Placebo		82	55.0	79.3	III (63)		APAH (37)	
Barst *et al*., 2011 (PHIRST-1b)^13^	Tadalafil	ERA	74	50.0	80	II (31)	16	IPAH (65)	RTI
	Placebo		37	51.7	78	III (67)		APAH (35)	
**sGC vs. Placebo**									
Ghofrani *et al*., 2013 (CHEST-1)^26^	Riociguat	Conventional therapy	173	59.0	68	II (31)	16	NA	RTI, SRTI
						III (64)			
	Placebo		88	59.0	61	IV (4)			
Ghofrani *et al*., 2013 (PATENT-1)^7^	Riociguat	PCA, ERA, or no	317	50.0	79.5	II (45)	12	IPAH (60)	RTI, SRTI
						III (52)		APAH (40)	
	Placebo		126	50.7	77.8	IV (1)			
Galiè *et al*., 2015 (PATENT PLUS)^27^	Riociguat	PDE5	12	58.0	50	II (56)	12	IPAH (50)	RTI, SRTI
	Placebo		6	61.0	50	III (33)		APAH (50)	
**Selective prostacyclin receptor agonist vs. Placebo**									
Simonneau *et al*., 2012^28^	Selexipag	ERA, or PDE5	33	54.8	81.8	II (40)	17	IPAH (81)	SRTI
	Placebo		10	53.8	80	III (60)		APAH (19)	
Sitbon *et al*., 2015 (GRIPHON)^8^	Selexipag	ERA, PDE5, both, or no	575	48.2	79.6	II (46)	71	IPAH (61)	RTI, SRTI
						III (53)		APAH (39)	
	Placebo		577	47.9	80.1	IV (1)			
**ERA vs. PDE5 vs. ERA+PDE5**									
Galiè *et al*., 2015 (AMBITION)^29^	Ambrisentan	Conventional therapy	152	53.9	79	II (31)	24	IPAH (59), APAH (41)	RTI, SRTI
	Tadalafil		151	54.5	83	III (69)			
	Ambrisentan + Tadalafil		302	54.5	74				

PCAs: prostanoids; ERAs: Endothelin receptor antagonists; PDE5s: Phosphodiesterase-5 inhibitors; sGCs: soluble guanylate cyclase simulators; PAH: pulmonary arterial hypertension; IPAH: idiopathic pulmonary arterial hypertension (includes familial or hereditary hypertension, or PAH due to drug or toxins and anorexigens); APAH: associated pulmonary arterial hypertension(includes PAH due to connective tissue disease, congenital heart disease, human immunodeficiency virus infection, and portal hypertension); CTEPH: chronic thromboembolic pulmonary hypertension; FIIP-PH: pulmonary hypertension associated with fibrotic idiopathic interstitial pneumonia; IPF-PH: pulmonary hypertension associated with idiopathic pulmonary fibrosis; WHO FC: World Health Organization functional class; NA: not available; N: number of patients; RTI: respiratory tract infection; SRTI: serious respiratory tract infection.

### Incidence of RTI and SRTI in PAH-specific drug therapy

As for RTI, 3579 patients in 19 RCTs treated with PAH-specific drugs were included in the analyses, and 713 (19.9%) of them experienced RTI events. A high incidence of 66.4% (89 of 134) was found in the PACES study^25^. Another four studies reported the RTI incidence ranging from 19.7% to 27.0%^8,22,23,29^. The AMBER I study showed the lowest incidence of 0% with none of the patients suffering from RTI^24^. As for SRTI, the analyses included 3582 patients in 20 RCTs receiving PAH-specific drugs, and 161 (4.5%) of them experienced SRTI events. The SRTI incidence was found over 10% in the PAH-specific drug therapy in three studies^21,22,25^, whereas no sign of SRTI events was reported in another 3 studies with the incidence being 0%^14,16,25^.

### Risk of RTI and SRTI compared with placebo

**Figure 2:**
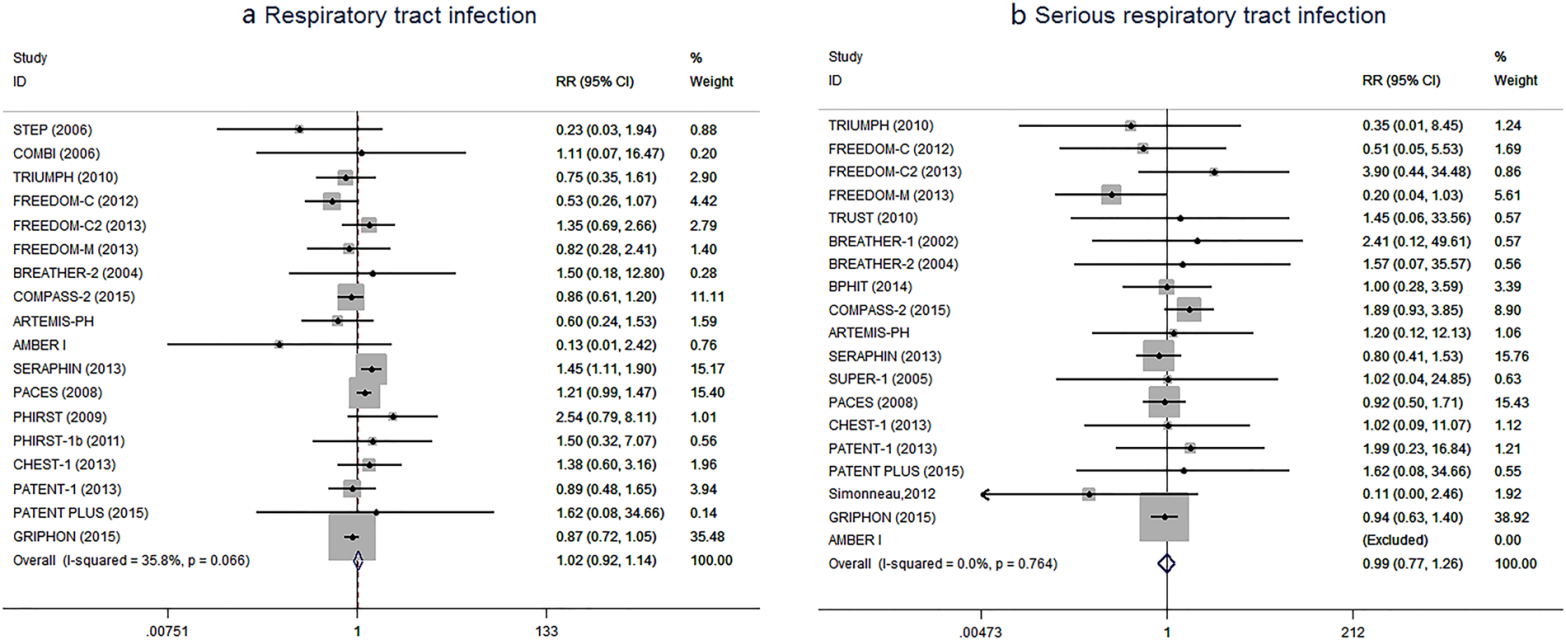
Forrest plot with meta-analysis of the risk of (**a**) Respiratory tract infection, and (**b**) serious Respiratory tract infection. RR indicates risk ratio. The size of data markers indicates the weight of each trial.

The overall effects of PAH-specific drugs for RTI and SRTI were presented on Fig. 2(a) and (b). Regarding RTI, 5065 PAH patients in 18 RCTs were incorporated when calculating the overall RR. In the PAH-specific drugs group, the incidence of RTI was 19.4% (576 of 2974), while that was 21.1% (442 of 2091) in the placebo group. The data failed to show a significantly higher risk with PAH-specific drugs than placebo (RR 1.02, 95%CI 0.92–1.14, *P* = 0.69), with no significant heterogeneity between included studies (*I* ^2^ *= *35.8%, *P* = 0.07). Regarding SRTI, 19 RCTs involving 5079 PAH patients were identified, and SRTI occurred with the incidence of 4.3% (129 of 2977) and 5.0% (105 of 2102) in PAH-specific drugs group and in the placebo group, respectively. The data showed that the use of PAH-specific drugs was not associated with significant risk increase compared with placebo (RR 0.99, 95%CI 0.77–1.26, *P* = 0.93), with no absence of heterogeneity between included studies (*I* ^2^ *= *0.0%, *P* = 0.76).

### Risk of RTI and SRTI based on the classes of PAH-specific drugs and therapy method

According to different classes of specific drugs, as shown in Table 2, all classes of PAH-specific drugs showed no significantly higher risk than placebo, which was concordant with the overall results. Similarly, no significant results were found based on therapy methods (Table 2). With respect to SRTI, as shown in Table 3, the results showed that no significant difference was detected regardless of different classes of specific drugs or therapy method.

**Table 2:** Subgroup analyses for respiratory tract infection.

**Treatment**	**No**. **of studies**	**With PAH-specific therapy**	**With placebo therapy**	**Total**	**RR**	**95%CI (p value)**	**Homogeneity**	
							**I** ^**2**^ **(%)**	**p value**
**Class of PAH-specific drugs**								
Prostanoids	6	49/651(7.5%)	58/579(10.0%)	107/1230(8.7%)	0.78	0.54–1.13(0.19)	0.8	0.41
ERAs	5	207/715(29.0%)	119/465(25.6%)	326/1180(27.6%)	1.14	0.93–1.40(0.20)	59.5	0.04
PDE5 inhibitors								
PACES^25^	1	89/134(66.4%)	72/131(55.0%)	161/265(60.8%)	1.21	0.99–1.47(0.06)	—	—
PHIRST^6^	1	30/323(9.3%)	3/82(3.7%)	33/405(8.1%)	2.54	0.79–8.11(0.12)	—	—
PHIRST-1b^13^	1	6/74(8.1%)	2/37(5.4%)	8/111(7.2%)	1.50	0.32–7.07(0.61)	—	—
sGCs	3	49/502(9.8%)	20/220(9.1%)	69/722(9.6%)	1.06	0.65–1.73(0.80)	0	0.68
Selective prostacyclin receptor agonist	1	146/575(25.4%)	168/577(29.1%)	314/1152(27.3%)	0.87	0.72–1.05(0.16)	—	—
**Monotherapy or combination therapy**								
Monotherapy vs. Placebo	4	33/366(0%)	21/196(1.5%)	1/129(0.8%)	0.86	0.51–1.44(0.57)	12.2	0.33
combination therapy vs. Monotherapy	15	605/2911(0%)	571/2499(2.3%)	3/262(1.1%)	0.99	0.90–1.10(0.91)	41.0	0.04

ERAs: Endothelin receptor antagonists; PDE5s inhibitors: Phosphodiesterase-5 inhibitors; sGCs: soluble guanylate cyclase simulators; RR: risk ratio.

**Table 3:** Subgroup analyses for serious respiratory tract infection.

Treatment	No. of studies	With PAH-specific therapy	With placebo therapy	Total	RR	95%CI (p value)	Homogeneity	
							**I** ^**2**^ **(%)**	**p value**
**Class of PAH-specific drugs**								
Prostanoids	5	8/627(1.3%)	9/540(1.7%)	17/1167(1.5%)	0.66	0.28–1.57(0.35)	20.4	0.29
ERAs	7	52/899(5.8%)	29/554(5.2%)	81/1453(5.6%)	1.20	0.78–1.83(0.40)	0	0.64
PDE5 inhibitors	2	18/341(5.3%)	18/201(9.0%)	36/542(6.6%)	0.93	0.51–1.70(0.81)	0	0.95
sGCs	3	8/502(1.6%)	2/220(0.9%)	10/722(1.4%)	1.54	0.38–6.26(0.55)	0	0.92
Selective prostacyclin receptor agonist	2	43/608(7.1%)	47/587(8.0%)	90/1195(7.5%)	0.90	0.61–1.33(0.60)	44.7	0.18
**Monotherapy or combination therapy**								
Monotherapy vs. Placebo	8	16/787(2.0%)	10/369(2.7%)	26/1156(2.2%)	0.76	0.37–1.56(0.45)	0	0.72
combination therapy vs. Monotherapy	12	139/2493(5.6%)	143/2337(6.1%)	282/4830(5.8%)	1.04	0.83–1.30(0.75)	0	0.71

ERAs: Endothelin receptor antagonists; PDE5s inhibitors: Phosphodiesterase-5 inhibitors; sGCs: soluble guanylate cyclase simulators; RR: risk ratio.

### Sensitivity Analyses

Sensitivity analysis, sequentially leaving each trial, was performed to assess the weight of each study in our analysis. The overall outcomes failed to identify any individual trials as having influenced the results of the present meta-analysis to a significant extent. Results of sensitivity analyses were consistent with those of the primacy analyses (Tables S3,S4).

### Publication Bias

Visual inspection of funnel plots for the analyses showed that all plots exhibited fairly symmetrical inverted funnel shapes, suggesting that publication bias was not a concern (Figure S5).

## Discussion

PAH is a hemodynamic abnormality common to a variety of conditions that is characterized by increased the afterload and work of right ventricle (RV), which ultimately leading to the failure of right heart^1,2^. Unlike left ventricle (LV), RV seems to be less able to adapt to pressure overload due to their differences in embryology, metabolism and vascularity^30^. Therefore, as an intercurrent illness of PAH, RTI can result in persistent hypoxia, increased heart oxygen consumption, inflammatory reaction, systematic oxidative stress, and unstable endothelial dysfunction, which may all contribute to increased workload of heart and subsequently lead to the deterioration of right heart failure^31–33^. This article is the first systematic review to pool current evidence for analyzing the risk of RTI in PAH patients with specific drug therapy, which combined evidence from 6307 PAH patients in 24 RCTs. The results indicated that the use of specific drugs in PAH did not significantly increase the risk of both RTI and SRTI when compared with placebo. The good robustness of the said results was substantiated in the finding of the sensitivity analyses. Meanwhile, the results of this study suggested that the negative effects on RTI and SRTI of PAH-specific drug therapy had little difference within different classes of drugs or therapy methods.

Regarding the incidence of RTI, we found that 19.9% of patients receiving PAH-specific drug therapy suffered from RTI. In the PACES trial, the incidence of RTI in sidenafil group and placebo group was 66.4% and 55.0%, respectively^25^. Whereas, in the AMBER I trial, no RTI was observed in ambrisentan group^24^. For SRTI, the results revealed that the incident was 4.5% in patients with specific drug therapy. In the COMPASS-2 trial, the incidence of SRTI in bosentan group and placebo was 11.9% and 6.3%, respectively^22^. Similarly, high SRTI rate was observed in the PACES trial comparing sildenafil (12.7% of SRTI) and placebo (13.7% of SRTI)^25^. On the opposite, no SRTI events were detected on specific drug therapy (treprostinil, ambrisentan, and selexipag) in 3 trials^16,24,28^. As the short duration of follow-up and intensive management in RCTs, RTI rate should be obtained in long-term observation study based on real-world experience.

On the background of high incidence of RTI in PAH with specific drug therapy, we assessed whether the specific therapy would be one of the contributing factors. The results confirmed that no significant increase in the risk of both RTI and SRTI was observed in PAH-specific drug therapy when compared to placebo.

Short-term RCTs investigating the effects of PAH-specific monotherapy have reported the improvements in haemodynamics and exercise capacity for PAH patients^26^. Nevertheless, long-term survival remained poor for PAH-specific monotherapy, with a mortality rate of 15% per year^34^. In an attempt to improve the prognosis and prolong the survival of PAH patients, combination therapy was proposed to modulate some various pathways of the disease at the same time. Recently, a meta-analysis including 4095 patients in 17 RCTs concluded that combination therapy for PAH was associated with a significant reduction in clinical worsening compared with monotherapy^35^. However, treatment discontinuation was more likely to occur in patients taking combination therapy^35^. A meta-analysis involving 6702 patients in 35 RCTs concluded that combination therapy showed a significant increase in the incidence of withdrawal due to adverse effects than monotherapy^36^. Based on the high incidence of adverse effects in combination therapy, the difference of RTI risk was evaluated between combination therapy and monotherapy. Our analyses revealed that combination therapy did not increase the risk of both RTI and SRTI compared to monotherapy.

RTI, as a precipitating factor, was independently associated with an incremental in-hospital mortality in patients with heart failure^37,38^. However, Diagnosing RTI is often challenging in patients admitted for heart failure due to similar symptoms and chest radiographs^39^. RTI, such as pneumonia, is commonly diagnosed on the basis of clinical features and demonstrable infiltrates on chest radiograph. Whereas, assessing pulmonary infiltration by means of chest radiograph may be hampered in heart failure patients because of pulmonary congestion. In addition, C reactive protein (CRP) is the most commonly used marker to aid the diagnosis of RTI. However, it may also indicate systemic inflammation independent of infections and it thus rather nonspecific. Heart failure is known to be an inflammatory status, and CRP may also be elevated due to the inflammatory state of heart failure alone^40^. Taken together, the diagnosis of RTI in patients with heart failure is really challenging. Unlike left heart failure, a clinical syndrome of right heart failure in PAH patients characterized by tissue congestion including jugular venous distention, peripheral edema, ascites, and abdominal organ engorgement. There is marked impairment of right ventricular systolic performance, usually with right ventricular dilatation and severe tricuspid regurgitation. In addition, CRP levels in PAH patients with right heart failure are rather low compared to those in RTI^41^. Except for CRP, specificity biomarker such as procalcitonin might help to resolve this uncertainty and improve antibiotic treatment strategy. RTI leads to a longer length of hospitalization and higher cost in PAH patients^41^. Although no increased RTI risk in PAH-specific drug therapy was observed when compared to placebo in the present study. RTI is worthy of clinical attention and intensive anti-infectious therapy should be considered for RTI in PAH patients.

Several important limitations were worth mentioned here. Firstly, 23 RCTs of specific drugs were excluded from the meta-analysis due to the RTI data unavailable, which might reduce the power of statistics. Secondly, the definition of RTI and SRTI was different across trials. Thirdly, we did not have access to data because of various etiology of PAH or World Health Organization functional class, making powerful subgroup analysis unavailable. Fourthly, different baseline therapy might influence the results. Fifthly, the observation time of the clinical trials included in our meta-analysis was inconsistent, from 12 to 71 weeks, which might also influence the results. Furthermore, none of included trials was especially designed to assess the safety of PAH-specific therapy. Therefore, RCTs focused on the safety of PAH-specific therapy and the long-term observation studies based on real-world experience are necessary to be conducted.

In conclusion, this is the first meta-analysis to assess the risk of RTI in PAH-specific drug therapy. The present study showed that specific drug therapy did not increase the risk of RTI in PAH. Whereas, RTI in PAH patients is still worthy of clinical attention and intensive anti-infectious treatment should be considered.

## Methods

### Data sources and searches

This systematic review and meta-analysis was reported in accordance with standards outlined in the Cochrane Handbook for Systematic Reviews of Intervention and the PRISMA Statement for Reporting Systemic Reviews and was conducted following a priori established protocol (PROSPERO: CRD42017064664)^42–44^. A comprehensive literature search of Medline, Embase, and Cochrane Library electronic databases was conducted to identify all potential eligible trials from inception to April 30, 2017 without language restriction. The following terms were used for searching: “pulmonary arterial hypertension” or “hypertension, pulmonary” or “pulmonary hypertension” or “PAH” in combination with “prostanoids” or “iloprost” or “treprostinil” or “epoprostenol” or “beraprost” or “endothelin receptor antagonists” or “bosentan” or “ambrisentan” or “macitentan” or “phosphodiesterase type 5 inhibitors” or “sildenafil” or “tadalafil” or “soluble guanylate cyclase stimulators” or “riociguat” or “selective prostacyclin receptor agonist” or “selexipag”. In addition, unpublished trials were identified from the ClinicalTrials.gov Website. The bibliographies of published trials and systematic reviews were also scrutinized to ensure that all relevant studies were identified. Two reviewers (Z.G. and C.Z.) independently searched the databases to identify all potential eligible studies, and all disagreements were resolved by consensus or by consulting a third author (X.L.).

### Study selection

Studies were involved if they met the following criteria. Only RCTs were included, and the participants should be adult patients with PAH. In addition, treatment had to involve PAH-specific drug therapy and reported the RTI or SRTI events for PAH-specific drugs and placebo, respectively. For multiple publications of the same RCT, we selected the publication most relevant to our inclusion criteria. Two reviewers (Z.G. and C.Z.) independently assessed all study titles and abstracts for determining eligibility, and full paper was retrieved and assessed when there was any possibility that it might be relevant. Regarding possible bias, Z.G. and C.Z. were blinded to authors’ names, journal names, and publication years of the papers. All discrepancies and uncertainties were resolved by consensus or by consulting a third author (X.L.).

### Data Extraction, quality evaluation and bias assessment

All data were extracted independently by two reviewers (Z.G. and C.Z.) using a priori designed form, including study population characteristics (first author’s name, publication year, sample size, mean age, sex, World Health Organization functional class, and etiology of PAH), PAH-specific therapy groups, comparison groups, background therapy, study duration, and the primacy outcome (RTI, or SRTI). RTI data that was not reported in the publications were further extracted from the ClinicalTrials.gov Website. Because RTI can be defined in various ways, and in order to ensure sufficient data for a meaningful analysis, the following adverse outcomes were used as RTI or SRTI, including upper respiratory tract infection, respiratory tract infection, pneumonia, bronchitis, lower respiratory tract infection, lung infection, and bronchopneumonia. The methodological quality of included RCTs was evaluated independently by Z.G. and C.Z. according to the Cochrane Collaboration Risk of Bias Tool, which include random sequence generation, allocation concealment, blinding, incomplete outcome data, selective reporting, and other bias^45^. The overall risk of bias was classified as low (all items were low risk, or at least 5 items were low risk and the remaining 2 unclear), unclear (>2 items were unclear risk), and high (≥1 items were high bias)^46^. Potential publication bias was evaluated by visually inspecting funnel plots, and would be minor if the plot of the magnitude of treatment effect in each study versus its precision estimate showed an approximate symmetrical funnel shape^42^.

### Data analysis

We carried out forest plots for measuring occurrence of RTI and SRTI, in which risk ratios (RRs) and their 95% CIs were calculated. The between-study heterogeneity was assessed through *I* ^2^ test that measures the percentage of total variation between studies, and a fixed-effects model was used based on Mantel-Haenszel method unless *I* ^2^ was >50%. All statistical analyses were performed by using STATA software (version13, Statacorp, College Station, Texas, USA), and *P* < 0.05 indicated a statistically significant difference.

Several included studies were 3 arms trial, and data on PAH-specific drugs was merged for these trials. For example, SERAPHIN was a 3-arm trial conducted on macitentan, which did not provide combined data versus placebo on RTI and SRTI^5^. Therefore, data of macitentan 3 mg group and macitentan 10 mg group was combined as a one camp. The same method was used in BREATHE-1^11^, SUPER-1^12^, PHIRST^6^, PHIRST-1b^13^, and PATENT-1^7^.

Sensitivity analyses were performed to identify the effect of a single trial by sequential elimination of each trial from the pool, and then to reassess the overall effects. Moreover, subgroup analyses, using a fixed-effects model, were also performed according to different class of specific drugs (PCAs, ERAs, PDE5 inhibitors, sGCs, and selective prostacyclin receptor agonist) and therapy method (monotherapy, or combination therapy).

### Study registration

PROSPERO Identifier, CRD42017064664.

## Electronic supplementary material

Supplementary information
