# Supplementary material for: Incidence and risk of respiratory tract infection associated with specific drug therapy in pulmonary arterial hypertension: a systematic review
Source: Sci Rep. 2017 Nov 24;7:16218. doi: 10.1038/s41598-017-16349-7 (PMC5701205; doi:10.1038/s41598-017-16349-7)
Supplement: Supplementary file 1 — Supplementary information [file 41598_2017_16349_MOESM1_ESM.pdf]

SUPPLEMENTAL FILE

Title: Incidence and risk of respiratory tract infection associated with specific drug therapy in pulmonary arterial hypertension: a systematic review

Authors: Zhichun Gu, Chi Zhang, Anhua Wei, Min Cui, Jun Pu, Houwen Lin, Xiaoyan Liu

Table S1. Randomized controlled trials excluded from the meta-analysis with reasons for exclusion

Study	Drugs	Drug class	Reason for exclusion
Rubin et al., 1990 [1]	Epoprostenol	IV/SC PCA	respiratory tract infection data unavailable
Barst et al., 1996 [2]	Epoprostenol	IV/SC PCA	respiratory tract infection data unavailable
Badesch et al., 2000 [3]	Epoprostenol	IV/SC PCA	respiratory tract infection data unavailable
Simonneau et al., 2002 [4]	Treprostinil	IV/SC PCA	respiratory tract infection data unavailable
McLaughlin et al., 2003 [5]	Treprostinil	IV/SC PCA	respiratory tract infection data unavailable
Oudiz et al., 2004 [6]	Treprostinil	IV/SC PCA	respiratory tract infection data unavailable
Bourge et al., 2016(DellVery)[7]	Treprostinil	IV/SC PCA	Single arm, and respiratory tract infection data unavailable
Olschewski et al., 2002 [8]	Iloprost	PO/INH PCA	respiratory tract infection data unavailable
Olschewski et al., 2010 [9]	Iloprost	PO/INH PCA	Single arm, and respiratory tract infection data unavailable
Saji et al., 2016[10]	Iloprost	PO/INH PCA	Single arm, and respiratory tract infection data unavailable
Galiè et al., 2002 (ALPHABET)[11]	Beraprost	PO/INH PCA	respiratory tract infection data unavailable
Barst et al., 2003 [12]	Beraprost	PO/INH PCA	respiratory tract infection data unavailable
Channick et al., 2001[13]	Bosentan	ERA	respiratory tract infection data unavailable
Galiè et al., 2006(BREATHE-5)[14]	Bosentan	ERA	respiratory tract infection data unavailable
Jaïs et al. 2008(BENEFIT)[15]	Bosentan	ERA	respiratory tract infection data unavailable
Galiè et al., 2008(EARLY)[16]	Bosentan	ERA	respiratory tract infection data unavailable
Galiè et al., 2008(ARIES-1,2)[17]	Ambrisentan	ERA	respiratory tract infection data unavailable
Barst et al., 2010(ASSET-1, 2)[18]	Bosentan	ERA	respiratory tract infection data unavailable
Wilkins et al., 2005[19]	Sildenafil, or bosentan	PDE5, ERA	respiratory tract infection data unavailable

Zhuang et al., 2014[20]	Tadalafil	PDE5	respiratory tract infection data unavailable
Vitulo et al., 2017[21]	Sildenafil	PDE5	respiratory tract infection data unavailable
Vizza et al., 2017[22]	Sildenafil	PDE5	Open label, and respiratory tract infection data unavailable
Bonderman et al., 2014(DILATE-1)[23]	Riociguat	sGC	respiratory tract infection data unavailable

PO/INH: oral or inhaled; IV/SC: intravenous or subcutaneous; PCA: prostanoid, ERA: Endothelin receptor antagonist; PDE5: Phosphodiesterase-5 inhibitor; sGC: soluble guanylate cyclase simulator.

Table S2. Quality assessment results of included randomized controlled trials.

Study	Random sequence generation	Allocation concealment	Blinding of participants and personnel	Blinding of outcome assessment	Incomplete outcome data	Selective reporting	Other bias	Summary bias
McLaughlin et al., 2006 (STEP) [24]	L	L	L	L	L	L	L	L
Hoeper et al., 2006 (COMBI) [25]	L	L	L	L	L	L	U	L
McLaughlin et al., 2010 (TRIUMPH) [26]	U	U	L	L	L	L	L	L
Tapson et al., 2012 (FREEDOM-C) [27]	L	U	L	L	L	L	L	L
Tapson et al., 2013 (FREEDOM-C2) [28]	L	U	L	L	L	U	L	L
Jing et al., 2013 (FREEDOM-M) [29]	L	U	L	L	L	L	L	L
Hiremath et al., 2010 (TRUST) [30]	L	L	U	L	L	L	L	U
Rubin et al., 2002(BREATHE-1)[31]	L	L	U	U	L	L	L	L
Humbert et al., 2004(BREATHE-2)[32]	L	L	U	U	L	L	L	L
Corte et al., 2014(BPHIT)[33]	U	U	U	U	L	L	L	U
McLaughlin et al., 2015 (COMPASS-2) [34]	U	U	L	L	L	L	L	L
ARTEMIS-PH [35]	U	U	U	U	L	L	U	U
AMBER I[36]	U	U	U	U	L	L	U	U
Pulido et al., 2013(SERAPHIN)[37]	L	L	L	L	L	L	L	L
Galiè et al., 2005 (SUPER-1)[38]	L	L	L	L	L	L	L	L
Simonneau et al., 2008 (PACES)[39]	L	L	L	L	L	L	L	L
Galiè et al., 2009 (PHIRST)[40]	U	U	L	L	L	L	L	L

Barst et al., 2011 (PHIRST-1b)[41]	U	U	L	L	L	L	L	L
Ghofrani et al., 2013 (CHEST-1)[42]	L	L	L	L	L	L	L	L
Ghofrani et al., 2013 (PATENT-1)[43]	L	L	L	L	L	L	L	L
Galiè et al., 2015 (PATENT PLUS)[44]	L	L	L	L	L	L	L	L
Simonneau et al., 2012[45]	L	U	L	L	L	L	L	L
Sitbon et al., 2015 (GRIPHON)[46]	L	L	L	L	L	L	L	L
Galiè et al., 2015 (AMBITION)[47]	L	L	L	L	L	L	L	L

L: low risk; U: unclear risk; H: high risk; The summary risk of bias was determined as low (all analyzed items were appropriate, or at least 5 items were appropriate and the remaining 2 unclear), unclear (>2 items were not reported), and high (⩾1 quality dimension suggested possible bias).

Table S3. Sensitivity analysis for respiratory tract infection

Study omitted	RR	95%CI
McLaughlin et al., 2006 (STEP) [24]	1.03	0.92-1.15
Hoeper et al., 2006 (COMBI) [25]	1.02	0.92-1.14
McLaughlin et al., 2010 (TRIUMPH) [26]	1.03	0.92-1.15
Tapson et al., 2012 (FREEDOM-C) [27]	1.05	0.94-1.17
Tapson et al., 2013 (FREEDOM-C2) [28]	1.01	0.91-1.13
Jing et al., 2013 (FREEDOM-M) [29]	1.03	0.92-1.14
Humbert et al., 2004(BREATHE-2)[32]	1.02	0.92-1.14
McLaughlin et al., 2015 (COMPASS-2) [34]	1.04	0.93-1.17
ARTEMIS-PH[35]	1.03	0.92-1.15
AMBER I[36]	1.03	0.92-1.15
Pulido et al., 2013(SERAPHIN)[37]	0.95	0.84-1.06
Simonneau et al., 2008 (PACES)[39]	0.99	0.87-1.12
Galiè et al., 2009 (PHIRST)[40]	1.01	0.90-1.12
Barst et al., 2011 (PHIRST-1b)[41]	1.02	0.92-1.14
Ghofrani et al., 2013 (CHEST-1)[42]	1.02	0.91-1.13
Ghofrani et al., 2013 (PATENT-1)[43]	1.03	0.92-1.15
Galiè et al., 2015 (PATENT PLUS)[44]	1.02	0.92-1.14
Sitbon et al., 2015 (GRIPHON)[46]	1.11	0.97-1.26

Table S4. Sensitivity analysis for serious respiratory tract infection

Study omitted	RR	95%CI
McLaughlin et al., 2010 (TRIUMPH) [26]	1.00	0.78-1.27
Tapson et al., 2012 (FREEDOM-C) [27]	1.00	0.78-1.28
Tapson et al., 2013 (FREEDOM-C2) [28]	0.96	0.75-1.23
Jing et al., 2013 (FREEDOM-M) [29]	1.04	0.81-1.33
Hiremath et al., 2010 (TRUST) [30]	0.99	0.77-1.26
Rubin et al., 2002(BREATHE-1)[31]	0.98	0.77-1.25
Humbert et al., 2004(BREATHE-2)[32]	0.99	0.77-1.26
Corte et al., 2014(BPHIT)[33]	0.99	0.77-1.27
McLaughlin et al., 2015 (COMPASS-2) [34]	0.90	0.69-1.17
ARTEMIS-PH[35]	0.99	0.77-1.26
AMBER I[36]	0.99	0.77-1.26
Pulido et al., 2013(SERAPHIN)[37]	1.03	0.79-1.34
Galiè et al., 2005 (SUPER-1)[38]	0.99	0.77-1.26
Simonneau et al., 2008 (PACES)[39]	1.00	0.77-1.31
Ghofrani et al., 2013 (CHEST-1)[42]	0.99	0.77-1.26
Ghofrani et al., 2013 (PATENT-1)[43]	0.98	0.76-1.25
Galiè et al., 2015 (PATENT PLUS)[44]	0.99	0.77-1.26
Simonneau et al., 2012[45]	1.01	0.79-1.29
Sitbon et al., 2015 (GRIPHON)[46]	1.02	0.75-1.39

Table S5. PRISMA Checklist

Section/topic	#	Checklist item	Reported on page #
**TITLE**			
Title	1	Identify the report as a systematic review, meta-analysis, or both.	1
**ABSTRACT**			
Structured summary	2	Provide a structured summary including, as applicable: background; objectives; data sources; study eligibility criteria, participants, and interventions; study appraisal and synthesis methods; results; limitations; conclusions and implications of key findings; systematic review registration number.	1,2
**INTRODUCTION**			
Rationale	3	Describe the rationale for the review in the context of what is already known.	3,4
Objectives	4	Provide an explicit statement of questions being addressed with reference to participants, interventions, comparisons, outcomes, and study design (PICOS).	3,4
**METHODS**			
Protocol and registration	5	Indicate if a review protocol exists, if and where it can be accessed (e.g., Web address), and, if available, provide registration information including registration number.	4
Eligibility criteria	6	Specify study characteristics (e.g., PICOS, length of follow-up) and report characteristics (e.g., years considered, language, publication status) used as criteria for eligibility, giving rationale.	4
Information sources	7	Describe all information sources (e.g., databases with dates of coverage, contact with study authors to identify additional studies) in the search and date last searched.	4
Search	8	Present full electronic search strategy for at least one database, including any limits used, such that it could be repeated.	4
Study selection	9	State the process for selecting studies (i.e., screening, eligibility, included in systematic review, and, if applicable, included in the meta-analysis).	5

Data collection process	10	Describe method of data extraction from reports (e.g., piloted forms, independently, in duplicate) and any processes for obtaining and confirming data from investigators.	5,6
Data items	11	List and define all variables for which data were sought (e.g., PICOS, funding sources) and any assumptions and simplifications made.	5,6
Risk of bias in individual studies	12	Describe methods used for assessing risk of bias of individual studies (including specification of whether this was done at the study or outcome level), and how this information is to be used in any data synthesis.	5,6
Summary measures	13	State the principal summary measures (e.g., risk ratio, difference in means).	6,7
Synthesis of results	14	Describe the methods of handling data and combining results of studies, if done, including measures of consistency (e.g., I^2) for each meta-analysis.	6,7

Section/topic	#	Checklist item	Reported on page #
Risk of bias across studies	15	Specify any assessment of risk of bias that may affect the cumulative evidence (e.g., publication bias, selective reporting within studies).	6,7
Additional analyses	16	Describe methods of additional analyses (e.g., sensitivity or subgroup analyses, meta-regression), if done, indicating which were pre-specified.	6,7
**RESULTS**			
Study selection	17	Give numbers of studies screened, assessed for eligibility, and included in the review, with reasons for exclusions at each stage, ideally with a flow diagram.	7,8
Study characteristics	18	For each study, present characteristics for which data were extracted (e.g., study size, PICOS, follow-up period) and provide the citations.	7,8
Risk of bias within studies	19	Present data on risk of bias of each study and, if available, any outcome level assessment (see item 12).	8
Results of individual studies	20	For all outcomes considered (benefits or harms), present, for each study: (a) simple summary data for each intervention group (b) effect estimates and confidence intervals, ideally with a forest plot.	8,9
Synthesis of results	21	Present results of each meta-analysis done, including confidence intervals and measures of consistency.	8,9
Risk of bias across studies	22	Present results of any assessment of risk of bias across studies (see Item 15).	8,9

Additional analysis	23	Give results of additional analyses, if done (e.g., sensitivity or subgroup analyses, meta-regression [see Item 16]).	10
**DISCUSSION**			
Summary of evidence	24	Summarize the main findings including the strength of evidence for each main outcome; consider their relevance to key groups (e.g., healthcare providers, users, and policy makers).	10,11,12
Limitations	25	Discuss limitations at study and outcome level (e.g., risk of bias), and at review-level (e.g., incomplete retrieval of identified research, reporting bias).	13
Conclusions	26	Provide a general interpretation of the results in the context of other evidence, and implications for future research.	13
**FUNDING**			
Funding	27	Describe sources of funding for the systematic review and other support (e.g., supply of data); role of funders for the systematic review.	14

From: Moher D, Liberati A, Tetzlaff J, Altman DG, The PRISMA Group (2009). Preferred Reporting Items for Systematic Reviews and Meta-Analyses: The PRISMA Statement. PLoS Med 6(6): e1000097. doi:10.1371/journal.pmed1000097

For more information, visit: **www.prisma-statement.org**.

Figure S1. Subgroup analyses for respiratory tract infection by Class of PAH-specific drugs

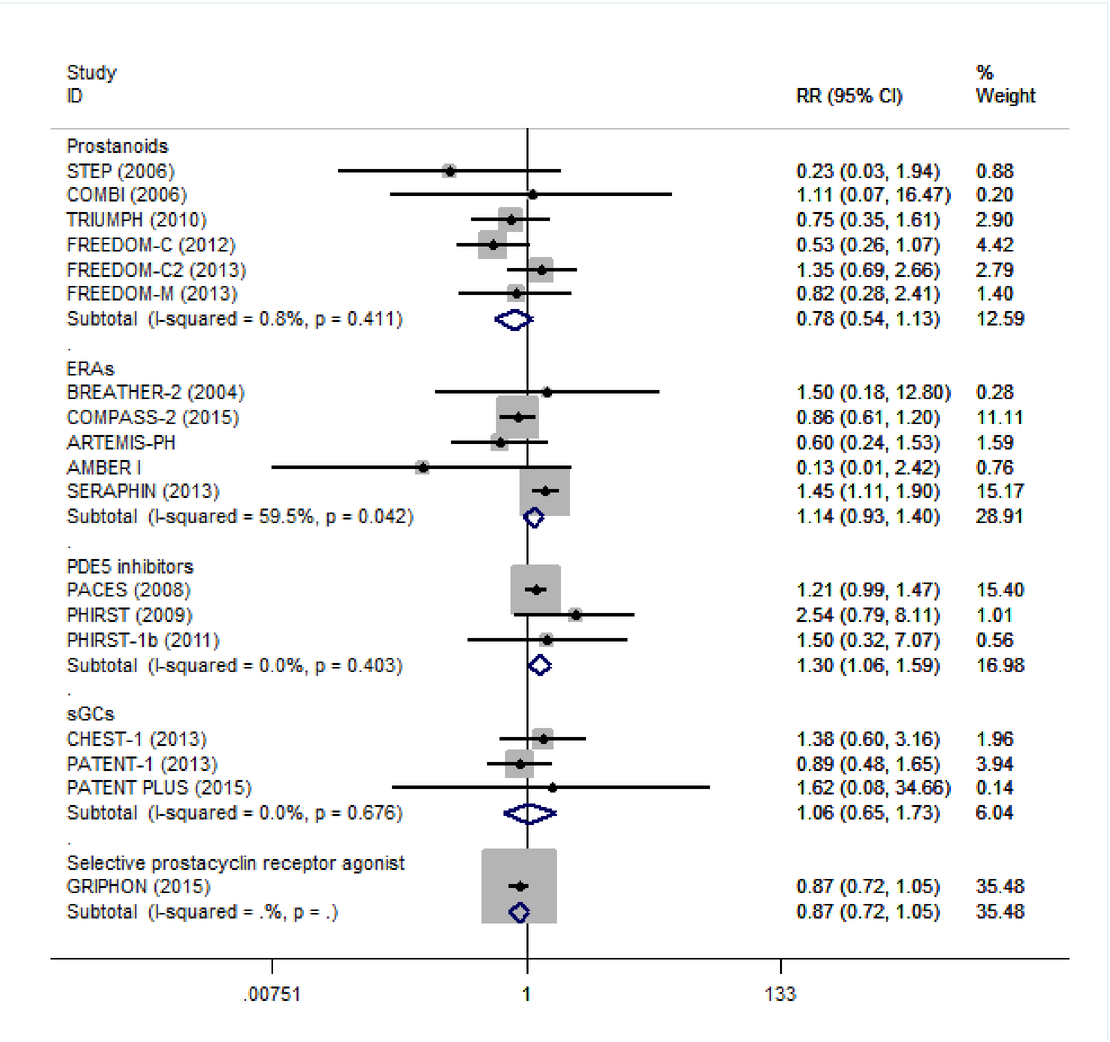

Figure S2. Subgroup analyses for respiratory tract infection by therapy method

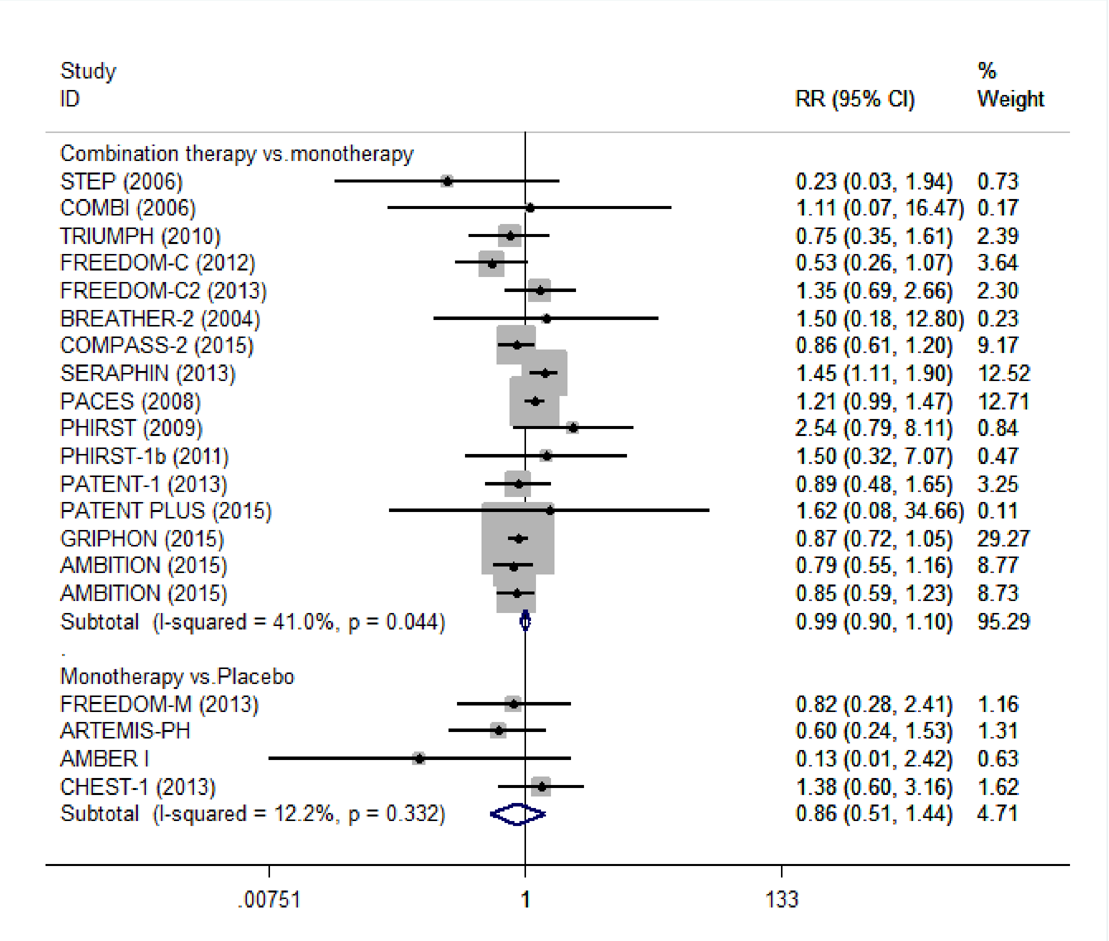

Figure S3. Subgroup analyses for serious respiratory tract infection by Class of PAH-specific drugs

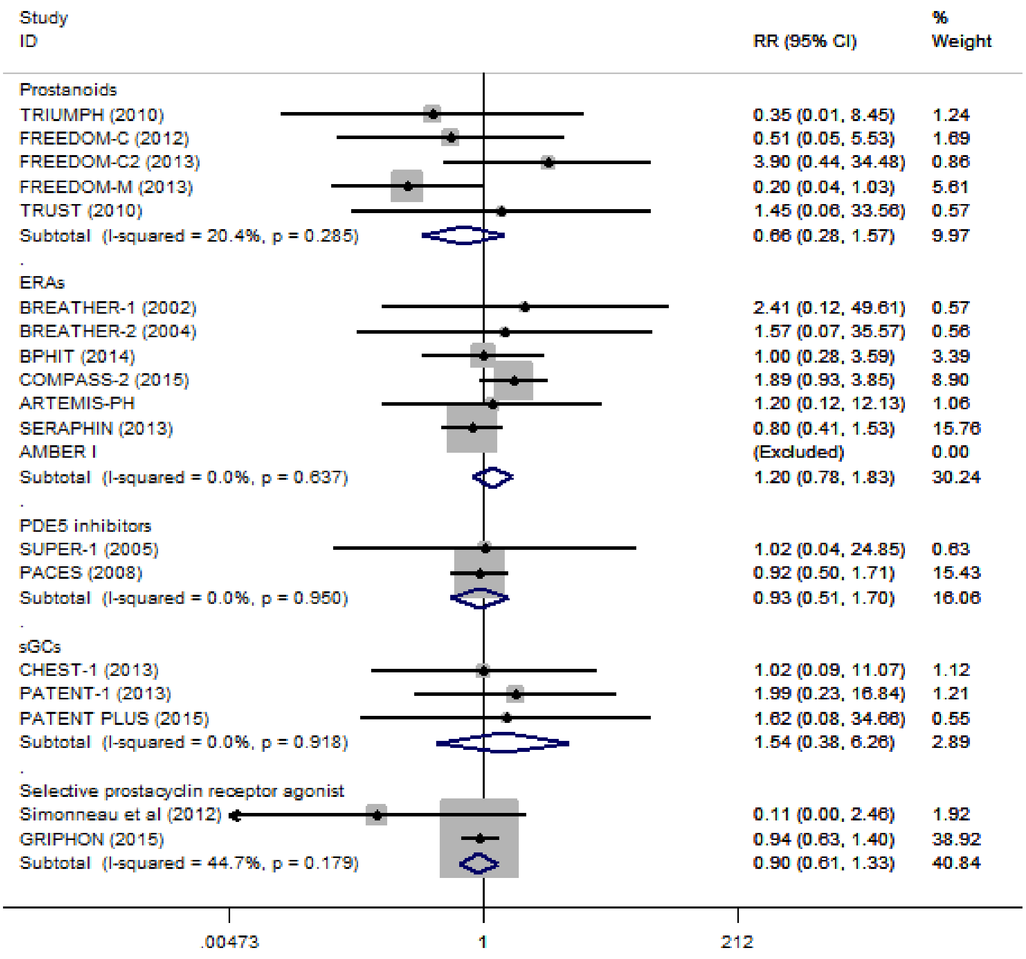

Figure S4. Subgroup analyses for serious respiratory tract infection by therapy method

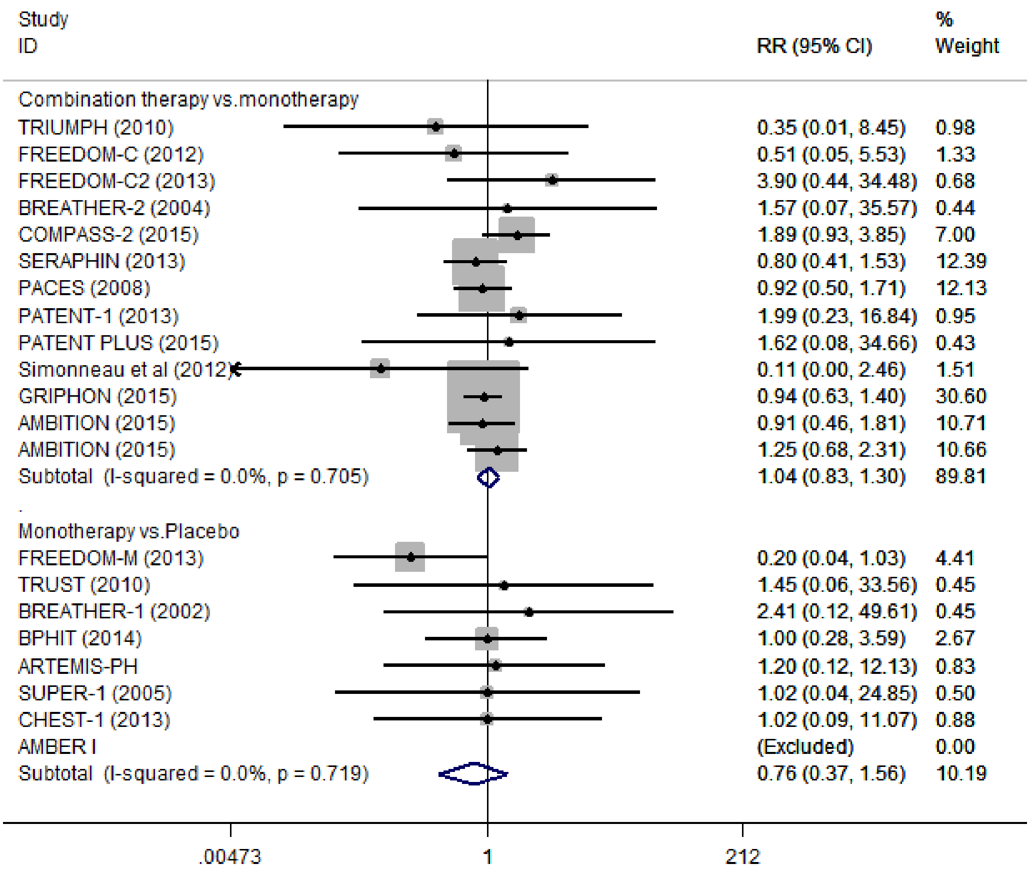

Figure S5. Funnel Plot of Studies Included in the Meta-analysis for (A) respiratory tract infection, and (B) serious respiratory tract infection

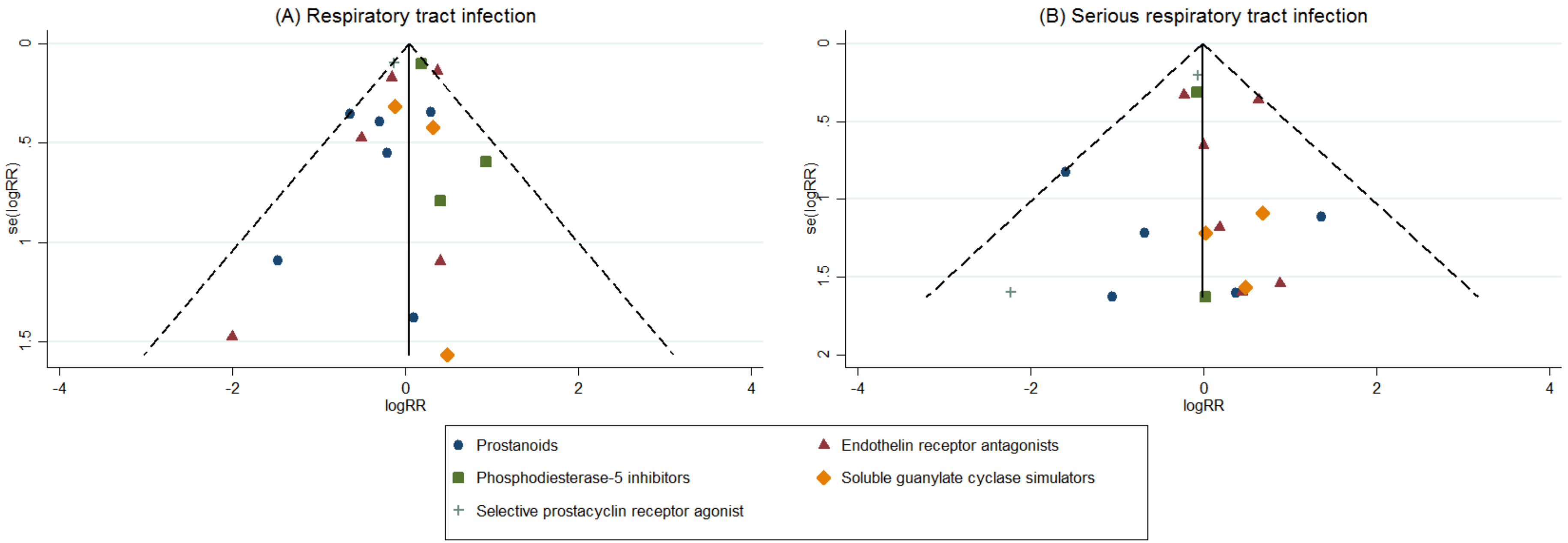

Legends

Tables

Table S1. Randomized controlled trials excluded from the meta-analysis with reasons for exclusion

Table S2. Quality assessment results of included randomized controlled trials

Table S3. Sensitivity analysis for respiratory tract infection

Table S4. Sensitivity analysis for serious respiratory tract infection

Table S5. PRISMA Checklist

Figures

Figure S1. Subgroup analyses for respiratory tract infection by Class of PAH-specific drugs

ERAs: endothelin receptor antagonists; PDE5 inhibitors: phosphodiesterase type 5 inhibitors; sGCs: soluble guanylate cyclase stimulators

Figure S2. Subgroup analyses for respiratory tract infection by therapy method

Figure S3. Subgroup analyses for serious respiratory tract infection by Class of PAH-specific drugs

ERAs: endothelin receptor antagonists; PDE5 inhibitors: phosphodiesterase type 5 inhibitors; sGCs: soluble guanylate cyclase stimulators

Figure S4. Subgroup analyses for serious respiratory tract infection by

therapy method

Figure S5. Funnel Plot of Studies Included in the Meta-analysis for (A) respiratory tract infection, and (B) serious respiratory tract infection

References

1. Rubin LJ, Mendoza J, Hood M, et al. Treatment of primary pulmonary hypertension with continuous intravenous prostacyclin (epoprostenol). Results of a randomized trial. *Ann Intern Med.* 1990;112:485-491.
2. Barst RJ, Rubin LJ, Long WA, et al. A comparison of continuous intravenous epoprostenol (prostacyclin) with conventional therapy for primary pulmonary hypertension. *N Engl J Med.* 1996;334:296-301.
3. Badesch DB, Tapson VF, McGoon MD, et al. Continuous intravenous epoprostenol for pulmonary hypertension due to the scleroderma spectrum of disease. A randomized, controlled trial. *Ann Intern Med.* 2000; 132:425-434.
4. Simonneau G, Barst RJ, Galie N, et al. Treprostinil Study Group. Continuous subcutaneous infusion of treprostinil, a prostacyclin analogue, in patients with pulmonary arterial hypertension: a double-blind, randomized, placebo-controlled trial. *Am J Respir Crit Care Med*. 2002;165:800-804.
5. McLaughlin VV, Gaine SP, Barst RJ, et al. Efficacy and safety of treprostinil: an epoprostenol analog for primary pulmonary hypertension. *J Cardiovasc Pharmacol.* 2003;41:293-299.
6. Oudiz RJ, Schilz RJ, Barst RJ, et al. Treprostinil, a prostacyclin analogue, in pulmonary arterial hypertension associated with connective tissue disease. *Chest.* 2004;126:420-427.
7. Bourge RC, Waxman AB, Gomberg-Maitland M, et al. Treprostinil Administered to Treat Pulmonary Arterial Hypertension Using a Fully Implantable Programmable Intravascular Delivery System: Results of the DelIVery for PAH Trial. *Chest.* 2016;150:27-34.

8. Olschewski H, Simonneau G, Galiè N, et al. Inhaled iloprost for severe pulmonary hypertension. *N Engl J Med.* 2002;347:322-329.

9. Olschewski H, Hoeper MM, Behr J, et al. Long-term therapy with inhaled iloprost in patients with pulmonary hypertension. *Respir Med.* 2010;104:731-740.

10. Saji T, Myoishi M, Sugimura K, et al. Efficacy and Safety of Inhaled Iloprost in Japanese Patients With Pulmonary Arterial Hypertension-Insights From the IBUKI and AIR Studies. *Circ J.* 2016; 80:835-842.

11. Galiè N, Humbert M, Vachiéry JL, et al. Effects of beraprost sodium, an oral prostacyclin analogue, in patients with pulmonary arterial hypertension: a randomized, double-blind, placebo-controlled trial. *J Am Coll Cardiol.* 2002;39:1496-1502.

12. Barst RJ, McGoon M, McLaughlin V, et al. Beraprost therapy for pulmonary arterial hypertension. *J Am Coll Cardiol.* 2003;41:2119-2125.

13. Channick RN, Simonneau G, Sitbon O, et al. Effects of the dual endothelin-receptor antagonist bosentan in patients with pulmonary hypertension: a randomised placebo-controlled study. *Lancet.* 2001;358:1119-1123.

14. Galiè N, Beghetti M, Gatzoulis MA, et al. Bosentan therapy in patients with Eisenmenger syndrome: a multicenter, double-blind, randomized, placebo-controlled study. *Circulation.* 2006;114:48-54.

15. Jaïs X, D'Armini AM, Jansa P, et al. Bosentan Effects in iNopErable Forms of chronIc Thromboembolic pulmonary hypertension Study Group. Bosentan for treatment of inoperable chronic thromboembolic pulmonary hypertension: BENEFiT (Bosentan Effects in iNopErable Forms of chronIc

Thromboembolic pulmonary hypertension), a randomized, placebo-controlled trial. *J Am Coll Cardiol*. 2008;52:2127-2134.

16. Galiè N, Rubin Lj, Hoeper M, et al. Treatment of patients with mildly symptomatic pulmonary arterial hypertension with bosentan (EARLY study): a double-blind, randomised controlled trial. *Lancet.* 2008;371:2093-2100.

17. Galiè N, Olschewski H, Oudiz RJ, et al. Ambrisentan in Pulmonary Arterial Hypertension, Randomized, Double-Blind, Placebo-Controlled, Multicenter, Efficacy Studies (ARIES) Group. Ambrisentan for the treatment of pulmonary arterial hypertension: results of the ambrisentan in pulmonary arterial hypertension, randomized, double-blind, placebo-controlled, multicenter, efficacy (ARIES) study 1 and 2. *Circulation*. 2008;117:3010-3019.

18. Barst RJ, Mubarak KK, Machado RF, et al. Exercise capacity and haemodynamics in patients with sickle cell disease with pulmonary hypertension treated with bosentan: results of the ASSET studies. *Br J Haematol.* 2010;149:426-435.

19. Wilkins MR, Paul GA, Strange JW, et al. Sildenafil versus Endothelin Receptor Antagonist for Pulmonary Hypertension (SERAPH) study. *Am J Respir Crit Care Med.* 2005;171:1292-1297.

20. Zhuang Y, Jiang B, Gao H, et al. Randomized study of adding tadalafil to existing ambrisentan in pulmonary arterial hypertension. *Hypertens Res.* 2014;37:507-512.

21. Vitulo P, Stanziola A, Confalonieri M, et al. Sildenafil in severe pulmonary hypertension associated with chronic obstructive pulmonary disease: A randomized controlled multicenter clinical trial. *J Heart Lung Transplant.*

2017;36:166-174.

22. Vizza CD, Sastry BK, Safdar Z,et al. Efficacy of 1, 5, and 20 mg oral sildenafil in the treatment of adults with pulmonary arterial hypertension: a randomized, double-blind study with open-label extension. *BMC Pulm Med.* 2017;17:44.

23. Bonderman D, Pretsch I, Steringer-Mascherbauer R, et al Acute hemodynamic effects of riociguat in patients with pulmonary hypertension associated with diastolic heart failure (DILATE-1): a randomized, double-blind, placebo-controlled, single-dose study. *Chest.* 2014;146:1274-1285.

24. McLaughlin VV, Oudiz RJ, Frost A, et al. Randomized study of adding inhaled iloprost to existing bosentan in pulmonary arterial hypertension. *Am J Respir Crit Care Med*. 2006; 174:1257-1263.

25. Hoeper MM, Leuchte H, Halank M, et al. Combining inhaled iloprost with bosentan in patients with idiopathic pulmonary arterial hypertension. *Eur Respir J.* 2006;28: 691-694.

26. McLaughlin VV, Benza RL, Rubin LJ, et al. Addition of inhaled treprostinil to oral therapy for pulmonary arterial hypertension: a randomized controlled clinical trial. *J Am Coll Cardiol*. 2010; 55:1915-1922.

27. Tapson VF, Torres F, Kermeen F, et al. Oral treprostinil for the treatment of pulmonary arterial hypertension in patients on background endothelin receptor antagonist and/or phosphodiesterase type 5 inhibitor therapy (the FREEDOM-C study): a randomized controlled trial. *Chest*. 2012; 142: 1383-1390.

28. Tapson VF, Jing ZC, Xu KF, et al. FREEDOM-C2 Study Team. Oral

treprostinil for the treatment of pulmonary arterial hypertension in patients receiving background endothelin receptor antagonist and phosphodiesterase type 5 inhibitor therapy (the FREEDOM-C2 study): a randomized controlled trial. *Chest*. 2013;144: 952-958.

29. Jing ZC, Parikh K, Pulido T, et al. Efficacy and safety of oral treprostinil monotherapy for the treatment of pulmonary arterial hypertension: a randomized, controlled trial. *Circulation.* 2013;127:624-633.

30. Hiremath J, Thanikachalam S, Parikh K, et al. Exercise improvement and plasma biomarker changes with intravenous treprostinil therapy for pulmonary arterial hypertension: a placebo-controlled trial. *J Heart Lung Transplant*. 2010; 29: 137-149.

31. Rubin LJ, Badesch DB, Barst RJ, et al. Bosentan therapy for pulmonary arterial hypertension. *N Engl J Med*. 2002; 346:896-903.

32. Humbert M, Barst RJ, Robbins IM, et al. Combination of bosentan with epoprostenol in pulmonary arterial hypertension: BREATHE-2. *Eur Respir J.* 2004; 24:353-359.

33. Corte TJ, Keir GJ, Dimopoulos K, et al. Bosentan in pulmonary hypertension associated with fibrotic idiopathic interstitial pneumonia. *Am J Resp Crit Care*. 2014; 190:208-217.

34. McLaughlin V, Channick RN, Ghofrani HA, et al. Bosentan added to sildenafil therapy in patients with pulmonary arterial hypertension. *Eur Respir J.* 2015; 46:405-413.

35. ARTEMIS-PH-study of ambrisentan in subjects with pulmonary hypertension associated with idiopathic pulmonary fibrosis. Available at:

https://www.clinicaltrials.gov/ct2/show/results/NCT00879229. Accessed January 10, 2017.

36. AMBER I-Ambrisentan for inoperable chronic thromboembolic pulmonary hypertension. Available at: https://clinicaltrials.gov/ct2/show/results/NCT01884675. Accessed January 10, 2017.

37. Pulido T, Adzerikho I, Channick RN, et al. Macitentan and morbidity and mortality in pulmonary arterial hypertension. *N Engl J Med.* 2013; 369:809-818.

38. Galiè N, Ghofrani HA, Torbicki A, et al. Sildenafil Use in Pulmonary Arterial Hypertension (SUPER) Study Group. Sildenafil citrate therapy for pulmonary arterial hypertension. *N Engl J Med.* 2005; 353: 2148-2157.

39. Simonneau G, Rubin LJ, Galiè N, et al. Addition of sildenafil to long-term intravenous epoprostenol therapy in patients with pulmonary arterial hypertension: a randomized trial. *Ann Intern Med.* 2008;149: 521-530.

40. Galiè N, Brundage BH, Ghofrani HA, et al. Pulmonary Arterial Hypertension and Response to Tadalafil (PHIRST) Study Group. Tadalafil therapy for pulmonary arterial hypertension. *Circulation.* 2009; 119: 2894-2903.

41. Barst RJ, Oudiz RJ, Beardsworth A, et al. Tadalafil monotherapy and as add-on to background bosentan in patients with pulmonary arterial hypertension. *J Heart Lung Transplant*. 2011; 30: 632-643.

42. Ghofrani HA, D'Armini AM, Grimminger F, et al. Riociguat for the treatment of chronic thromboembolic pulmonary hypertension. *N Engl J Med.* 2013; 369: 319-329.

43. Ghofrani HA, Galiè N, Grimminger F, et al. Riociguat for the treatment of

pulmonary arterial hypertension. *N Engl J Med.* 2013; 369: 330-340.

44. Galiè N, Müller K, Scalise AV, et al. PATENT PLUS: a blinded, randomised and extension study of riociguat plus sildenafil in pulmonary arterial hypertension. *Eur Respir J*. 2015; 45: 1314-1322.

45. Simonneau G, Torbicki A, Hoeper MM, et al. Selexipag: an oral, selective prostacyclin receptor agonist for the treatment of pulmonary arterial hypertension. *Eur Respir J.* 2012; 40:874-880.

46. Sitbon O, Channick R, Chin KM, et al. Selexipag for the Treatment of Pulmonary Arterial Hypertension. *N Engl J Med*. 2015; 373: 2522-2533.

47. Galiè N, Barberà JA, Frost AE, et al. AMBITION Investigators. Initial Use of Ambrisentan plus Tadalafil in Pulmonary Arterial Hypertension. *N Engl J Med*. 2015; 373: 834-844.
